# Altering under-represented DNA sequences elevates bacterial transformation efficiency

**DOI:** 10.1128/mbio.02105-23

**Published:** 2023-10-31

**Authors:** Shuai Hu, Stefani Giacopazzi, Ryan Modlin, Kevin Karplus, David L. Bernick, Karen M. Ottemann

**Affiliations:** 1 Department of Microbiology and Environmental Toxicology, University of California, Santa Cruz, California, USA; 2 Department of Biomolecular Engineering, University of California, Santa Cruz, California, USA; University of Illinois Chicago, Chicago, Illinois, USA

**Keywords:** transformation, restriction enzyme, genomes

## Abstract

A cornerstone of bacterial molecular biology is the ability to genetically manipulate the microbe under study. Many bacteria are difficult to manipulate genetically, a phenotype due in part to robust removal of newly acquired DNA, for example, by restriction-modification (R-M) systems. Here, we report approaches that dramatically improve bacterial transformation efficiency, piloted using a microbe that is challenging to transform due to expression of many R-M systems, *Helicobacter pylori*. Initially, we identified conditions that dampened expression of several R-M systems and concomitantly enhanced transformation efficiency. We then identified an approach that would broadly protect newly acquired DNA. We computationally predicted under-represented short DNA sequences in the *H. pylori* genome, with the idea that these sequences reflect targets of sequence-based surveillance such as R-M systems. We then used this information to modify and eliminate such sites in antibiotic resistance cassettes, creating a “stealth” version. Modifying antibiotic resistance cassettes in this way resulted in significantly higher transformation efficiency compared to non-modified cassettes, a response that was genomic loci independent. Our results suggest that avoiding R-M systems, via modification of under-represented DNA sequences or transformation conditions, is a powerful method to enhance DNA transformation. Our approach to identify under-represented sequences is applicable to any microbe with a sequenced genome.

**IMPORTANCE:**

Manipulating the genomes of bacteria is critical to many fields. Such manipulations are made by genetic engineering, which often requires new pieces of DNA to be added to the genome. Bacteria have robust systems for identifying and degrading new DNA, some of which rely on restriction enzymes. These enzymes cut DNA at specific sequences. We identified a set of DNA sequences that are missing normally from a bacterium’s genome, more than would be expected by chance. Eliminating these sequences from a new piece of DNA allowed it to be incorporated into the bacterial genome at a higher frequency than new DNA containing the sequences. Removing such sequences appears to allow the new DNA to fly under the bacterial radar in “stealth” mode. This transformation improvement approach is straightforward to apply and likely broadly applicable.

## INTRODUCTION

Bacterial genetic manipulation is an important tool for modern bacteriology and underlies the growing desire to manipulate diverse microbes, e.g., from polluted environments, the human microbiome, with goals such as to create new probiotics, living drugs, or superbugs that can clean up toxic waste or act as biofertilizers (1). To realize these goals, a wide variety of bacteria must be manipulated efficiently.

One barrier that currently limits genetic manipulation is robust host restriction-modification (R-M) systems that digest newly acquired DNA. R-M systems use a restriction endonuclease (REase) and a methyltransferase (MTase) (2). Each acts on specific DNA sequences. The REase typically cleaves unmethylated DNA at the sequence, and the methyltransferase methylates the same sequence to block the REase (3). There are numerous R-M systems, recognizing hundreds of sequences (4). Some bacterial species express dozens of R-M systems. One of these is *Helicobacter pylori*, a naturally competent human pathogen that expresses up to 26 R-M systems (5).

Because R-M systems limit genetic manipulation, several strategies have been developed to diminish REase cleavage. One strategy is to methylate the exogenous DNA *in vitro* using purified MTases or a crude extract from the target bacterium (6–8). This approach is low-cost and can be applied to many microbes, but has challenges since not all MTases are available in pure forms and crude extracts contain competing DNases or variable MTase activity (9). Another strategy is to subvert the restriction-modification activities by either stimulating endogenous MTases or eliminating the REases via genetic engineering (10, 11). REases have been predicted based on homology to known REases, or based on presence of methylated genomic sequences, but these approaches can be time consuming if there are many REases.

REases operate by binding specific DNA sequences that are typically four to six basepairs long. Having these sites present on the genome has some risks, because they could be cut when in the non- or hemi-methylated form, e.g., after DNA replication. Thus, it would be evolutionarily advantageous to avoid REase sites in the genome. Indeed, Rocha et al. reported that palindromes with half sites of size 4 to 6 bases, potential REase targets, are under-represented in some prokaryotic genomes (12). Here, we expand this concept to identify all under-represented short DNA sequences, not only palindromes, because many REases, e.g., type I, type III, and type IV, recognize non-palindromic sequences (13–15). We developed a method to predict under-represented motifs in the genome sequence. We then used this information to create modified antibiotic resistance cassettes that lacked these sequences but retained the original encoded amino acids. We report here that the efficiency of the transformation was increased greatly in *H. pylori*, suggesting this method may have broad applications to improve efficient genetic manipulation.

## RESULTS

### Low R-M system expression improves *H. pylori* natural transformation

**Fig 1:**
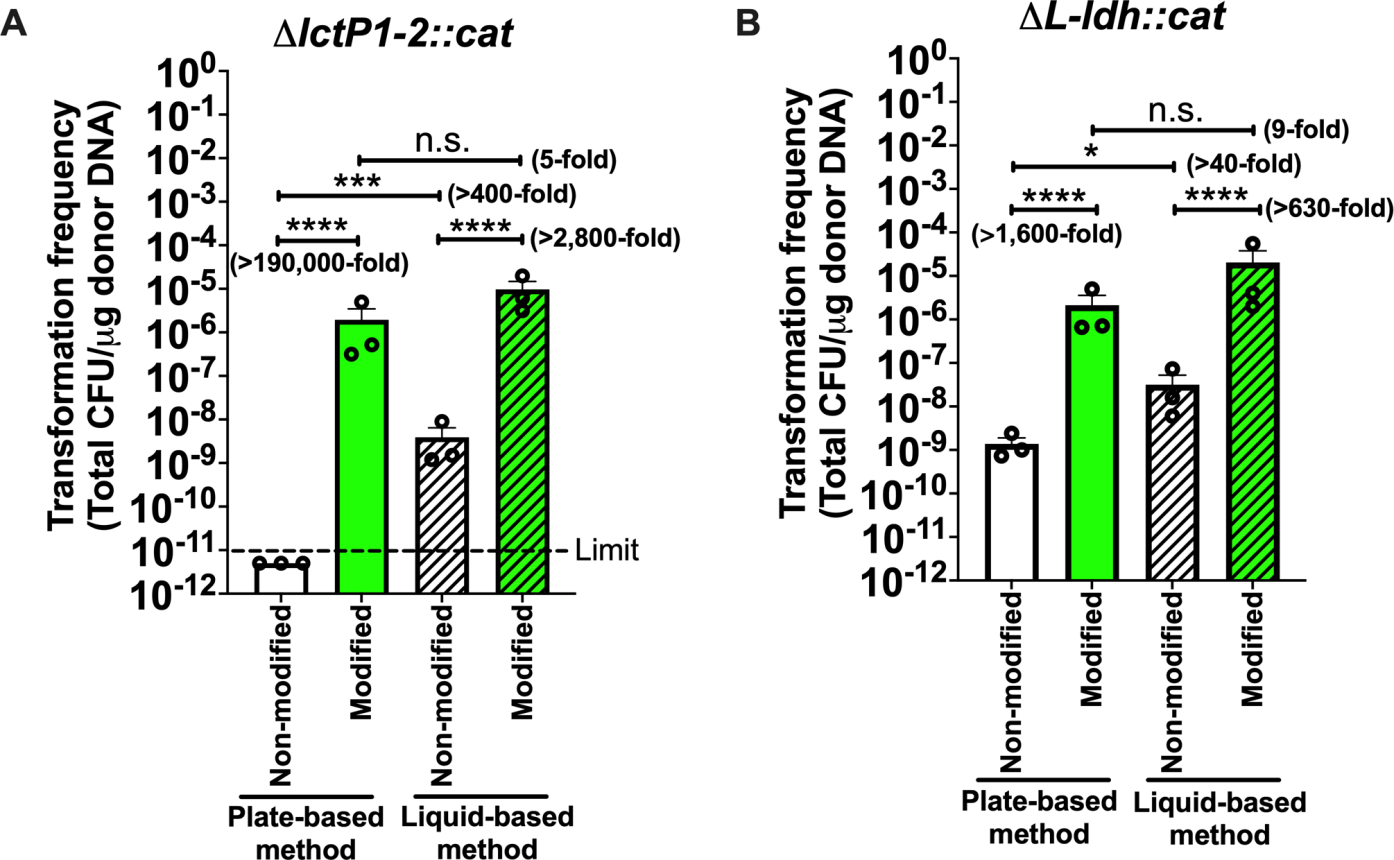
Transformation conditions and engineered antibiotic cassettes significantly improve *H. pylori* transformation efficiency. *H. pylori* pre-mouse Sydney Strain 1 (PMSS1) was transformed with 5 µg of the indicated PCR product. Transformation efficiency was measured by determining the number of chloramphenicol-resistant CFU divided by the total CFU, per microgram of DNA. Target gene transformation efficiencies were compared between conditions for (**A**) ∆*lctP1-2::cat* and (**B**) ∆*L-ldh::cat* cassettes. Results are presented as mean transformation efficiency ± standard error of the mean. Three technical triplicates of each sample were averaged to give a value for one biological replicate, and each biological replicate graphed as an open circle; log scaling not done for analysis, but only for plotting. The *P*-values were obtained with a one-way analysis of variance with the Tukey *post hoc* test. The significance is indicated as * (*P* < 0.05), *** (*P* < 0.001), **** (*P* < 0.0001), or n.s. (not significant).

*H. pylori* expresses multiple R-M systems (5), and these drastically limit transformation efficiency (16). Some *H. pylori* restriction enzyme genes are expressed to high levels in biofilm growth versus planktonic conditions (17, 18). Normal transformations employ plate-grown cells, which may mimic a biofilm state, so we wondered whether using planktonic conditions would affect transformation. We targeted the *lctP* locus (*lctP 1-2*, *hp0140-0141*) (19, 20), creating a construct that flanked a chloramphenicol resistance (Cm^R^) gene *cat* (21) with *lctP1* upstream and *lctP2* downstream sequences, called ∆*lctP1-2::cat* (Fig. S1). Using plate-grown *H. pylori*, we were unable to obtain any transformants with 5 µg of linear ∆*lctP1-2::cat* DNA. The transformation efficiency was calculated by dividing the number of Cm^R^ colony forming units (CFUs) by the total CFU, per microgram of DNA. This experiment yielded a transformation efficiency <10^−11^ CFU per microgram (Fig. 1A). Incorporating a liquid-based step, in contrast, elevated the transformation efficiency to 10^−9^ CFU per microgram DNA (Fig. 1A). This result suggests that transformation under low-REase expression conditions can promote *H. pylori* transformations.

### Under-represented short DNA sequences can be identified in the *H. pylori* genome and used to modify an antibiotic resistance cassette

Given the marked transformation improvement obtained in low-REase activity conditions, we further investigated whether creating antibiotic cassettes that evade REase recognition would promote transformation. We explored the idea that DNA sequences under-represented in the genome might be REase recognition sites. We screened the *H. pylori* genome for short DNA sequences that occur significantly less frequently than would be expected, using a Markov chain to model the expected frequencies for all DNA sequences of four to eight bases, called Kmers, in the genome. Kmers of size 4, for example, were modeled with a Markov (2) model and Kmers of size six were modeled with a Markov (4) model. Formally, this technique is equivalent to the use of marginal frequencies in a contingency table to produce expected values (the null) for comparison with observed frequencies. In both cases, prediction of an expected sequence relies on the frequencies of the subsequences that make up the larger modeled Kmer of interest. We then approximated parameters of a normal distribution (mean and SD) approximated from the binomial distribution to calculate Z-scores for observed frequencies, and applied a Bonferroni-adjusted Z-score cutoff of −6.7. We applied this analysis to the *H. pylor*i Sydney Strain 1 (SS1) genome (22) and found that there were multiple under-represented sequences; the top 42 had significant Z-scores (Table S1).

**Fig 2:**
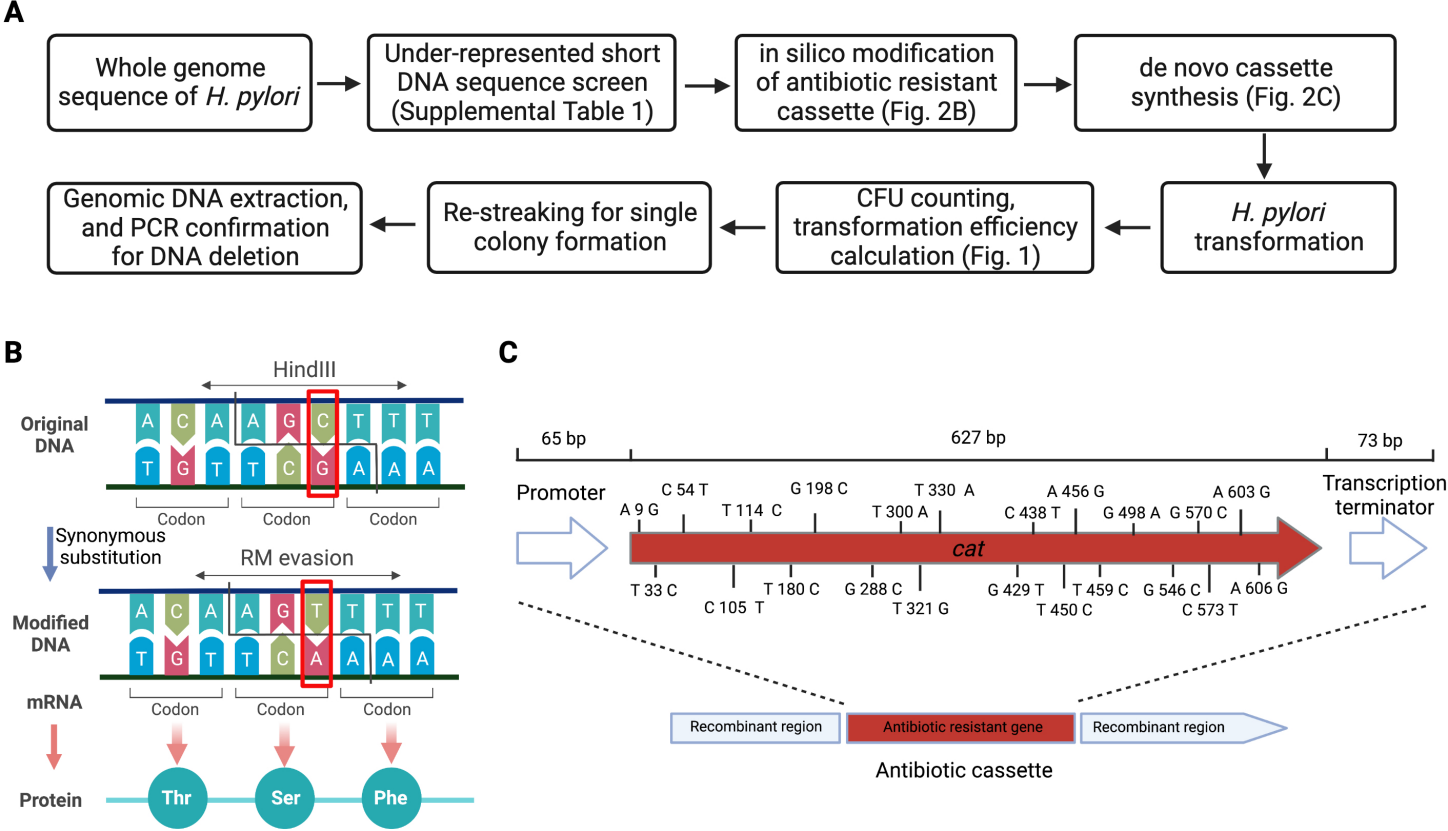
Schematic representation of approach to identify and modify under-represented sequences. (**A**) Workflow. (**B**) Example strategy of gene modification. The gene (*cat*) sequence was compared against the predicted under-represented motifs list (Table S1), and bases within these sequences were changed with synonymous substitutions at the wobble position. (**C**) Schematic of the modified cat gene (*cat*_stealth_), with 22 nucleotides synonymously substituted based on under-represented sequence prediction.

We next created a version of the *cat* chloramphenicol resistance cassette that lacked the 42 under-represented sites within the coding sequence (Fig. 2), changing 22 predicted under-represented sequences using synonymous mutations that did not alter the amino-acid sequence (Fig. 2C; Fig. S2). This modified cassette is referred to as *cat*_stealth_.

### Transformation efficiency is significantly elevated with stealth antibiotic cassettes

Next, we evaluated whether the modified *cat*_stealth_ cassette gained improved transformation efficiency compared to wild-type (WT) *cat*. We used the same *lctP* locus as above, replacing WT *cat* with *cat*_stealth_ (Fig. S1). Using the plate-based method, transformation efficiency increased from undetectable to 1.9 × 10^−6^ per microgram DNA with *cat*_stealth_, a >10^5^-fold improvement (Fig. 1A). This result suggests that removal of possible restriction sites has a dramatic effect on transformation efficiency.

We next examined whether the modified cassette would enhance transformation of other loci. We chose the L-lactate dehydrogenase coding genes (*L-ldh*, *hp0137-0139*) (19), to create ∆*L-ldh::cat*. The WT *cat* cassette yielded 10^−9^ CFU per microgram DNA with the plate-based method (Fig. 1B), a level that was different from the *lctP* locus, suggesting these two locations act independently. Transformation with the *cat*_stealth_ version resulted in a 1,600-fold increase to 10^−6^ CFU per microgram (Fig. 1B). These outcomes suggested that targeting under-represented sequences works in several loci.

We further tested whether transformation with the modified cassette also enhanced transformation in liquid, low-REase conditions. In these conditions, *cat*_stealth_ enhanced transformation of both *lctP* by 2,800- fold (Fig. 1A) and *L-ldh* by 630-fold (Fig. 1A and B). These results suggest that removing under-represented DNA sequences from antibiotic-resistant cassettes can enhance transformation under multiple experimental conditions.

## DISCUSSION

In this study, we report an efficient approach to improve microbial genetic engineering by facilitating R-M system evasion. The bulk of this approach was based on the speculation that genomes evolve to eliminate restriction sites, resulting in under-representation of these sequences. We used this idea to identify under-represented sequences in a target bacterial genome, and then mutated them in an antibiotic resistance cassette. This approach is relatively simple and resulted in transformation efficiency that increased by several orders of magnitude.

We reported two approaches that improved transformation. The liquid-based transformation method uses an environment that naturally lowers *H. pylori* REase expression, inspired by the observation that the HypAV, HpyAIV, and R.Pab1 REases are expressed to lower levels in planktonic conditions versus biofilm (17, 18). Using conditions skewed toward planktonic yielded a ~100- to 1,000-fold increased transformation efficiency in *H. pylori* PMSS1, a strain well known to be challenging to transform.

We obtained the most substantial transformation improvement, however, by modifying the incoming DNA to lack under-represented genomic sequences. A similar incoming DNA modification strategy was reported in by Johnston et al. in *Staphylococcus aureus* (11). These authors identified possible REase sites experimentally, based on single-molecule real-time genome and methylome sequencing. They inferred that the methylated motifs would be those recognized by active REases, and modified them. They similarly reported a five order of magnitude transformation efficiency improvement. Our approach, in contrast, identified potential REase recognition motifs by screening under-represented short DNA sequences. The approach of Johnston et al. requires several different types of sequencing, while our approach needs only the whole genome sequence of the target bacterium. We envision that the approach reported here will facilitate highly feasible genetic manipulation in a broad range of microbes.

## SUPPLEMENTAL MATERIAL

Supplemental material mbio.02105-23-s0001.docx Supplemental methods, figures, and tables.

ASM does not own the copyrights to Supplemental Material that may be linked to, or accessed through, an article. The authors have granted ASM a non-exclusive, world-wide license to publish the Supplemental Material files. Please contact the corresponding author directly for reuse.
