# Supplementary material for: Altering under-represented DNA sequences elevates bacterial transformation efficiency
Source: mBio. 2023 Oct 31;14(6):e02105-23. doi: 10.1128/mbio.02105-23 (PMC10746208; doi:10.1128/mbio.02105-23)
Supplement: Supplemental material — Supplemental methods, figures, and tables. [file mbio.02105-23-s0001.docx]

## Supplemental Materials

## Materials and Methods

### Strains, and growth conditions

*H. pylori* wild-type strain PMSS1 (Arnold et al., 2012) and *E. coli* DH10B (NEB) were used in this study (Supplemental Table 2). *H. pylori* strains were grown on Colombia Horse Blood Agar containing 5% defibrinated horse blood (Hemostat Laboratories, Dixon, CA), 0.2% β-cyclodextrin, 10 µg/ml vancomycin, 5 µg/ml cefsulodin, 2.5 U/ml polymyxin B, 5 µg/ml trimethoprim, and 8 µg/ml amphotericin B (CHBA) (all chemicals are from Thermo Fisher or Gold Biotech). For liquid media, *H. pylori* cultures were grown in Brucella Broth (BB) medium supplemented with 10% heat-inactivated fetal bovine serum (FBS) (BB10) (Life Technologies). *H. pylori* were grown under microaerobic conditions of 10% CO_2_, 5% O_2_ and 85% N_2_ at 37°C. *E. coli* DH10B strain was used to maintain de novo synthesized non-modified (WT) or modified cat (*cat*_stealth_) cassettes. The stock *E. coli* strains were maintained in Luria-Bertani (LB) broth plus 30% final concentration of glycerol (Fisher) in -80 °C. For liquid culture, each *E.coli* stain was inoculated in LB broth and grown in aerobic condition at 37°C.

### Under-represented Kmers analysis

We used an order (n-2) Markov model as the null model to evaluate an arbitrary n-mer, presented as a word (W), that occurs significantly less frequently than expected. This approach is naturally elegant for even-length palindromes. For the odd-length palindromes, we assumed a degenerate letter (N) in the middle. To compute the expected probability (P) of word(W), we used the observed frequencies of the lefthand and righthand k-1 mers along with the central k-2mer.

When expressed as probabilities,

$$P\left( W \right)=\frac{{P(W}_{1}..W_{k-1}){P(W}_{2}..W_{k})}{{P(W}_{2}..W_{k-1})}$$

We then estimate the expected count(C) of W, using the actual counts of the three component kmers, yielding:

$$E\left( C\left( W \right) \right)=\frac{{C(W}_{1}..W_{k-1}){C(W}_{2}..W_{k})}{{C(W}_{2}..W_{k-1})}$$

We further modeled the distribution of expected counts as binomial in order to assign a probability to the observed count of W:

$$\mu=genomeSize\times P\left( W \right)$$

$$Var=genomeSize\times P\left( W \right)\times\left( 1-P\left( W \right) \right)$$

The Z-score was then computed for the observed count of W, using:

$$Z\left( W \right)=\frac{C\left( W \right)-\mu}{\sqrt{Var}}$$

A Bonferroni multiple hypothesis correction was applied to our Zscore cutoff to select significantly under-represented, reverse-complement palindromic Kmers for this study by dividing the p-value associated with our Z-score cutoff by the number of tests applied to this dataset ( 96 in this case; 4mers (16), 5mers (16) 6mers (64)).

### Code for prediction of under-represented Kmer sequences

The code for this software was developed in Python and remains in active development as of this writing. *H. pylori* SS1 genome sequence in FASTA format was downloaded from UCSC genome browser database^23^. The code, named Stealth v0, can be found at <https://git.ucsc.edu/dbernick/Stealth>.

### Construction of modified antibiotic resistant gene

The 42 most significantly underrepresented Kmers were collected (Supplemental Table 1). To facilitate finding these motif sequences in the target genes, we matched each sequence to a restriction enzyme, and then used common restriction enzyme finders in Geneious (Biomatters) to find those motif sequences in the *Campylobacter coli* *cat* (Supplemental Table 2). After finding the sequences, we manually edited each sequence to alter the third codon base to eliminate the restriction site without altering the amino acid produced, except in rare cases where the amino acids were changed to conserved residues.

### Creation of antibiotic resistant cassettes

To *in silico* assemble the antibiotic resistant cassette for *lctP1-2* targeted transformation, 500 basepairs upstream of *lctP1* (*hp0140*) and 500 basepairs downstream of *lctP*2 (*hp0141*) were flanked with isogenic *cat* genes, WT and *cat*_stealth_, respectively (Supplemental figure 1). The cassettes were then commercially synthesized (Biobasic), and maintained in pUC59 plasmid vectors, called pUC59-*cat*_wt_ or pUC59-*cat*_stealth_, (Supplemental Table 2). For longterm preservation, plasmids were transformed into *E. coli* DH10B strain and stored in -80 °C in 15% glycerol. For *L-ldh* targeted transformation, 500 basepairs upstream of *hp0137* and 500 basepairs downstream of *hp0139* were PCR amplified and fused with either WT *cat* or *cat*_stealth_, (Supplemental Table 2) (Supplemental Figure 1).

### Transformation assays

For the classical plate-based method, a large loop of 1-day-old *H. pylori* from a CHBA plate was inoculated onto a new CHBA plate for 5 hours. 5 µg of PCR product with the desired antibiotic cassette was added to the top of the cultured bacteria. The mixture was incubated on the plate for 18-20h in microaerobic conditions.

For the liquid-based method, a large loop of 1-day-old *H. pylori* from a CHBA plate was resuspended in 200ul BB10 and incubated for 1h under static, microaerobic conditions. 5 µg of PCR product with desired antibiotic cassette was added to the resuspended *H. pylori* culture. The tubes were incubated for 5 minutes at room temperature, then the entire amount transferred to a CHBA plate for additional 18-20 hours incubation in microaerobic condition.

After the incubation period, the *H. pylori* was collected from the plate and resuspended in BB10. The sample was serially diluted followed by plating on selective plates with Cm or CHBA plates. Plates were incubated in microaerobic conditions for 4-6 days to allow bacterial colonies formation. Transformation efficiency for each experiment was calculated as (CFU × dilution factor on selective plate)/(CFU × dilution factor on CHBA plate)/5µg DNA.

## Supplemental Tables and Figures


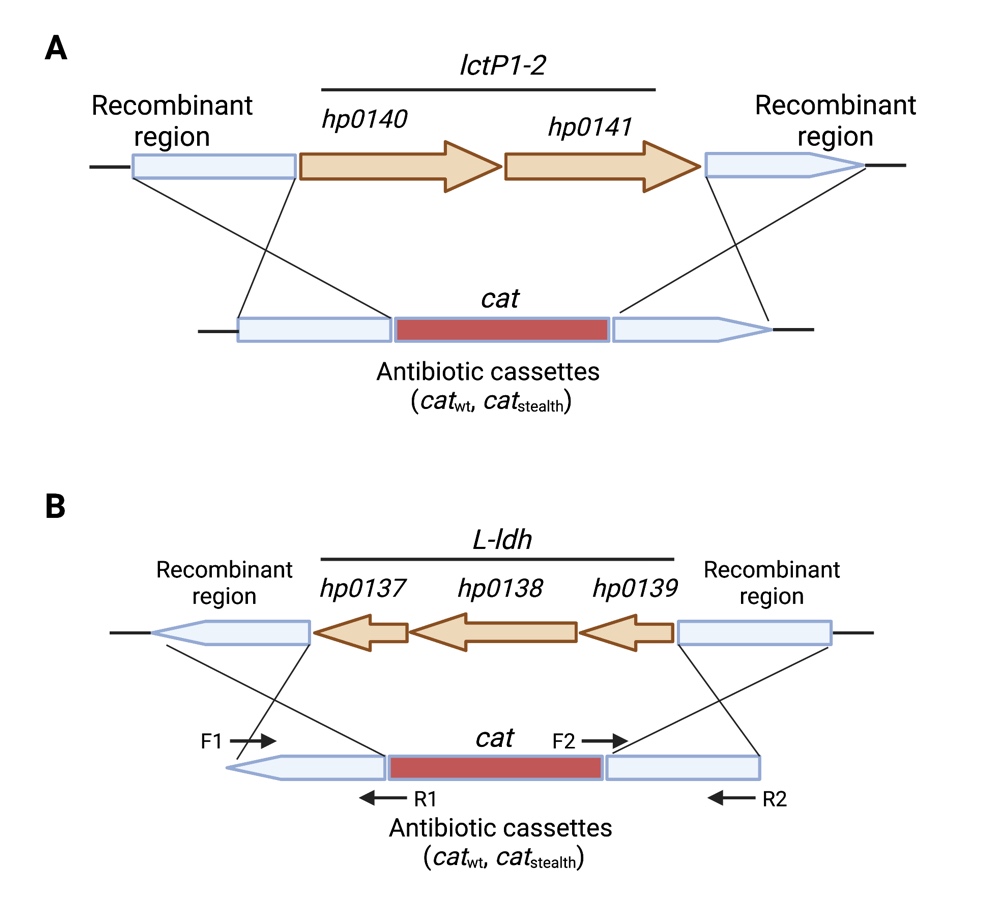


### Supplemental Figure 1 Schematic of recombination based gene deletion in *H. pylori*

(A) Schematic of *H. pylori* transformations, which occur by double cross overs. (A) *lctP1-2* or (B) *L-ldh*. Primer annealing locations are shown for panel B.

tatgatatagtggatagatttatgatataatgagttatcaacaaatcggcatctacggaggataaATGATGCAATTTACAAAGATTGATATAAATAACTGGACACGAAAAGAGTATTTTGACCACTATTTTGGCAATACGCCCTGTACATATAGTATGACAGTAAAACTAGATATTTCGAAGTTGAAAAAGGACGGAAAAAAGTTATACCCAACTCTTTTATATGGAGTGACAACGATAATCAACCGACATGAAGAGTTCAGAACCGCATTAGATGAAAACGGACAGGTAGGCGTTTTTTCTGAAATGCTGCCTTGCTACACCGTTTTTCATAAGGAAACTGAAACCTTTTCAAGTATTTGGACCGAGTTCACAGCAGACTATACCGAGTTTCTCCAGAACTATCAAAAGGATATAGACGCTTTTGGCGAACGAATGGGAATGTCCGCAAAGCCTAATCCTCCGGAAAACACTTTCCCTGTTTCTATGATACCATGGACAAGTTTTGAAGGCTTCAACTTGAATCTAAAAAAAGGATATGACTATCTACTGCCGATATTTACATTTGGGAAGTATTATGAGGAGGGCGGAAAATACTATATTCCCTTATCAATTCAAGTACATCATGCCGTTTGCGACGGCTTTCATGTTTGCCGTTTTTTGGATGAGTTACAAGACTTGCTGAATAAATAAaatcccagtttgtcgcactgataaaaaccctttaggaactaaagggcacacttctatactctctgccgagagtagtgcgtcctgcgga

### Supplemental Figure 2 Modified *cat*_stealth_ sequence

Lower case indicates upstream and downstream transcription promoter or terminator regions; pper case indicates coding sequence.

| Kmer | observed | expected | Zscore | Enzyme |
| --- | --- | --- | --- | --- |
| GATATC | 30 | 400.99 | -18.53 | EcoRV |
| GAATTC | 178 | 622.95 | -17.83 | EcoRI |
| GCGCGC | 278 | 666.57 | -15.05 | Uba69I |
| GAGCTC | 94 | 286.44 | -11.37 | SacI |
| GTTAAC | 29 | 180 | -11.26 | HpaI |
| GCTAGC | 266 | 505.2 | -10.64 | BmtI |
| TCTAGA | 158 | 351.08 | -10.31 | XbaI |
| CGCGCG | 116 | 282.13 | -9.89 | None |
| TCGCGA | 11 | 111.01 | -9.49 | SpoI |
| CCTAGG | 71 | 185.97 | -8.43 | BlnI |
| GGCGCC | 32 | 114.83 | -7.73 | KasI |
| GTGCAC | 43 | 130.31 | -7.65 | DaqI |
| TAATTA | 344 | 515.37 | -7.55 | PacI (TTAATTAA) |
| GTATAC | 15 | 81.2 | -7.35 | SnaI |
| CCATGG | 224 | 363.19 | -7.3 | NcoI |
| GGATCC | 107 | 206.1 | -6.9 | BamHI |
| TCATGA | 282 | 420.8 | -6.77 | BspHI |
| GANTC | 2737 | 5249.07 | -34.73 | HinFI |
| ACNGT | 564 | 2062.51 | -33.02 | HpyF121 |
| CTNAG | 3008 | 5279.1 | -31.31 | HpyF11I |
| GCNGC | 2476 | 3693.16 | -20.05 | Fsp4HI |
| GGNCC | 861 | 1617.05 | -18.81 | Sau96I |
| TCNGA | 1308 | 2032.63 | -16.08 | Hpy188I |
| CGNCG | 1291 | 1944.19 | -14.82 | Hpy991 (CGWCG) |
| CANTG | 4558 | 5234.25 | -9.36 | None |
| CCNGG | 1425 | 1732.8 | -7.4 | HpyNI |
| AGNCT | 5969 | 6562.63 | -7.34 | None |
| AANTT | 15939 | 16855.93 | -7.1 | None |
| GTNAC | 1707 | 2021.92 | -7.01 | MaeIII |
| ACGT | 211 | 3091.73 | -51.86 | HpyF13III |
| CGCG | 2606 | 6054.01 | -44.4 | HpyF52II |
| TCGA | 311 | 2461.92 | -43.38 | TaqI |
| GCGC | 6157 | 10103.61 | -39.39 | HhaI |
| AGCT | 6682 | 10366.18 | -36.3 | AluI |
| GGCC | 1569 | 3789.42 | -36.11 | HaeIII |
| GTAC | 132 | 1173.79 | -30.42 | RsaI |
| ATAT | 5803 | 7225.01 | -16.77 | None |
| CTAG | 4199 | 5431.75 | -16.75 | Rma485I |
| AATT | 14412 | 16288.11 | -14.77 | TseCI |
| GATC | 5263 | 6208.08 | -12.02 | BstXI |
| TTAA | 18389 | 19667.64 | -9.17 | MseI |

### Supplemental Table 1 Under-represented DNA sequences in *H. pylori* strain SS1.

DNA sequences that are underrepresented in the *H. pylori* SS1 genome. Observed indicates the number of times this sequence appeared in the genome; expected is the number of times this sequence was predicted. The Zscore indicates the confidence that the indicated Kmer is underrpresented. Enzymes column indicates an enzyme that cuts this site, and was used as a tool to locate the sequences in the target genes. If the enzyme cut a slightly different site, that is indicated in parentheses.

| **Strain** | **Genotype or description** | **Reference and/or source(s)** |
| --- | --- | --- |
| *H. pylori* PMSS1 | WT strain | Arnold et al., 2011 |
| *Campylobacter coli* | WT *cat gene* | Wang et al., 1990 |
| PMSS1  *ΔlctP1-2:: cat*_wt_ | *Δhp0140-0141::cat*_wt_ | This study (KO1797) |
| PMSS1 *Δlctp1-2::cat*_stealth_ | *Δhp0140-0141::cat*_stealth_ | This study (KO1710) |
| PMSS1 *ΔL-ldh:: cat*_wt_ | *Δhp0137-0139::cat*_wt_ | This study (KO1793) |
| PMSS1 *ΔL-ldh::cat*_stealth_ | *Δhp0137-0139::cat*_stealth_ | This study (KO1794) |
| *E. coli* DH10B | #3019I | NEB |
| DH10B pUC59-*cat*_wt_ | *Δhp0140-0141::cat*_wt_ | This study (KO1795) |
| DH10B pUC59-*cat*_stealth_ | *Δhp0140-0141::cat*_stealth_ | This study (KO1796) |

### Supplemental Table 2 Strains and plasmids used in this study

### Supplemental Table 3 Primers for generation of *L-ldh*-*cat* cassettes

| Primer name | Sequence |
| --- | --- |
| Primer 1 | TTAGTAGAGAATGATAGCGACTTTTTGAGGC |
| Primer 2 | TCTGCCGAGAGTAGTGCGTCCTGCGGAATTGAAGCGATGCGTGAAGAGTTGTTT |
| Primer 3 | ATTATATCATAAATCTATCCACTATATCATACATGTTGAATTCCGCATGCCCTTCAAACAA |
| Primer 4 | TTGAAAGTCAATTTCTTTGCTACTTGTCT |

*************************************************************************
